# DIFFERENTIAL DIGITAL DIVIDES: AGE GAPS ACROSS EXISTING AND EMERGING TECHNOLOGIES

**DOI:** 10.1093/geroni/igae098.4161

**Published:** 2024-12-31

**Authors:** Edie Sanders, George Mois, Wendy Rogers, Walter Boot

**Affiliations:** Weill Cornell Medicine, New York, New York, United States; University of Georgia, Athens, Georgia, United States; University of Illinois Urbana-Champaign, Champaign, Illinois, United States; Weill Cornell Medicine, New York, New York, United States

## Abstract

As the necessity of digital technology and the number of devices needed to participate in everyday life increases, understanding age differences in the use of familiar and emerging technologies is crucial for implementing technology-based solutions to enhance independence and wellbeing. We conducted an online survey to understand the types of technology categories used across age groups to support everyday life. Forty younger adults (aged 18-35 years), 40 middle-aged adults (35-64 years), and 40 older adults (65+ years) reported technology use across 13 categories (e.g., smart phones, robots) within the past year. Age-related differences in technology use varied across technology categories. For example, nearly every participant in each age group reported using a computer, a navigation device, a smartphone, and a TV, devices commonly used for everyday activities. Several technologies, including virtual reality and wearable devices, were reported most frequently by middle-aged adults. The use of tablets was reported least frequently by younger adults, voice-activated assistants were reported most frequently by older adults, and gaming consoles were reported less frequently with each increasing age group. Robots were less commonly used, and this pattern was similar for all age groups. These data challenge the idea that older adults are overall less likely to adopt and use technology compared to younger age groups. Understanding technology use at the level of an individual may be more important than at the level of age group for tailoring technology interventions that support everyday activities.

